# Characterizing Changes in Screen Time During the COVID-19 Pandemic School Closures in Canada and Its Perceived Impact on Children With Autism Spectrum Disorder

**DOI:** 10.3389/fpsyt.2021.702774

**Published:** 2021-08-18

**Authors:** Robyn E. Cardy, Annie Dupuis, Evdokia Anagnostou, Justine Ziolkowski, Elaine A. Biddiss, Suneeta Monga, Jessica Brian, Melanie Penner, Azadeh Kushki

**Affiliations:** ^1^Bloorview Research Institute, Holland Bloorview Kids Rehabilitation Hospital, Toronto, ON, Canada; ^2^Division of Biostatistics, Dalla Lana School of Public Health, University of Toronto, Toronto, ON, Canada; ^3^Department of Paediatrics, University of Toronto, Toronto, ON, Canada; ^4^Institute of Biomedical Engineering, University of Toronto, Toronto, ON, Canada; ^5^Department of Psychiatry, University of Toronto, Toronto, ON, Canada; ^6^Department of Psychiatry, Hospital for Sick Children, Toronto, ON, Canada

**Keywords:** autism spectrum disorder, screen time, COVID-19, pandemic, children

## Abstract

The COVID-19 pandemic has led to an increase in screen time for children and families. Traditionally, screen time has been associated with negative physical and mental health outcomes, and children with autism spectrum disorder (ASD) are at increased risk of these outcomes. The primary objectives of this study were to (1) characterize the change in screen time during COVID-19 school closures for children with ASD, and (2) examine the parent perceived impact of screen time on mental health and quality of life of children and their families. Canadian parents and caregivers of children 19 years of age and younger were eligible to participate in an anonymous, online survey study. This survey was available in English, consisted of 28 questions, took ~10-min to complete, and was available for 6 weeks (May 22 through July 6, 2020). The total sample consisted of 414 responses (ASD: *n* = 127, mean age = 11.7 ± 4.06 years; community sample: *n* = 287, mean age = 9.4 ± 4.26 years). Seventy-one respondents were missing responses to our primary question and removed from the analyses (final sample *n* = 344). Compared to the community sample, the ASD group had a significantly higher screen time use before and during the COVID-19 pandemic school closures [weekdays: difference = 1.14 (SE = 0.18), *t* = 6.56, *p* < 0.0001; weekends: difference = 1.41 (SE = 0.20), *t* = 6.93, *p* < 0.0001]. Mean total screen time during the pandemic was 6.9 h (95% CI 6.49, 7.21) on weekdays and 6.3 h (95% CI 5.91, 6.63) on weekends for the ASD group, and 5.6 h (95% CI 5.28, 5.92) on weekdays and 5.0 h (95% CI 4.70, 5.34) on weekends for the community sample. There was a significant increase in screen time during the COVID-19 pandemic as compared to before the pandemic period in the ASD group [weekdays: mean difference = 3.8 h (95% CI 3.35–4.25), *p* < 0.0001; weekends: mean difference = 1.5 h (95% CI 1.17–1.92), *p* < 0.0001]. Gender was a significant predictor of parent perceived mental health and quality of life, with male gender associated with a higher likelihood of negative impact [quality of life (child/family) OR = 1.8 (95% CI 1.1–2.9), corrected *p* = 0.040; mental health OR = 1.9 (95% CI 1.1–3.1), corrected *p* = 0.0028]. Parents' most frequently endorsed emotions toward screen time were guilt, frustration, and worry. Results of this survey study revealed that children with ASD were less likely to benefit from screen time to cope with social isolation, and screen time resulted in significantly more lost time on social interactions than the community sample, which may exacerbate difficulties in social domains. Given the unprecedented circumstances of the COVID-19 pandemic and the novel context of technology use, the findings of this study highlight the need for revision of screen time recommendations to reflect the current needs of children and families.

## Introduction

School closures, social distancing, and other pandemic response measures introduced to control the spread of the novel coronavirus disease (COVID-19) have led to a massive surge in screen time for children and youth (1–4). Screen time, defined as the amount of time interacting with electronic screen technology, is traditionally associated with a multitude of negative physical and mental health outcomes. However, the novel context of technology use during the pandemic period is challenging the traditional notions of harm as many families now depend on digital devices to cope with the imposed restrictions and psychological impact of the pandemic. Screen time, which is typically associated with social isolation (5, 6), is now paradoxically enabling virtual social connections to mitigate the impact of physical and social distancing requirements. Often viewed as hindering academic activities (7), screen time is quickly becoming the primary mode of education for many children. And social media, which is thought to contribute to anxiety and depression (8–13) and misinformation (14) is also becoming a place to seek support, share positive messaging, and disseminate COVID-19 resources and strategies to improve mental health.

Despite its significant influence on physical and mental health, the impact of screen time on children and youth has been largely neglected in the current pandemic and very little is known about how much screen time use has changed during this period. Of specific concern, even less is known about screen time experiences of children and youth with autism spectrum disorder (ASD), who are at increased risk of adverse outcomes from screen time (15, 16). ASD is a complex neurodevelopmental disorder characterized primarily by differences in social communication skills and the presence of restricted/repetitive behaviors (17), with a prevalence of 1 in 66 (15.2 per 1,000) children aged 5–17 years in Canada (18). Prior to the COVID-19 pandemic, children with ASD were exposed to more screen time compared to typically developing (TD) children and other clinical groups (19). The observed greater and earlier onset of interest in screen viewing in this population is likely influenced by a multitude of factors [see systematic review by (19)], such as the amount of social, cognitive, or physical effort required, the interpersonal reprieve it may provide, and the lack of barriers to engagement. Higher pre-pandemic screen time, combined with the loss of services and supports during the pandemic and the disorder's hallmark pre-disposition for intense and restricted pre-occupations (17), may put children and youth with ASD at a greater risk than other children for negative outcomes of increased screen time during the COVID-19 pandemic. Given the shift in the context of use and increased reliance on technology during the pandemic, there is a critical need to understand the both the changes in screen time use and its impact on the well-being and quality of life (QoL) of children with ASD.

This study aims to fill the above knowledge gaps by surveying screen time use in Canadian children and youth with ASD during the school closures following the pandemic declaration on March 11, 2020. Our primary objectives were to: (1) characterize the change in parent reported screen time in children with ASD during the COVID-19 school closures, and (2) examine the parent perceived impact of screen time on mental health and QoL of children with ASD and their families. Our exploratory aims were to examine predictors of screen time change and perceived impact.

## Materials and Methods

### Research Design

An anonymous online parent survey was created and managed using REDCap electronic data capture tools hosted at Holland Bloorview, Canada's largest pediatric rehabilitation hospital located in Toronto, Ontario (20, 21). The survey was disseminated through email distribution lists and/or social media channels (Twitter, Facebook) belonging to Holland Bloorview, the Province of Ontario Neurodevelopmental Network (22), and autism organizations and research centers. The survey was available for ~6 weeks (May 22 through July 6, 2020) covering the end of the final term of the 2019–2020 school year in Canada. All participants provided electronic informed consent. Upon completion of the survey, participants were able to enter a draw for one of three $50 gift cards. The study was approved by the institution's Research Ethics Board.

### Participants

Canadian parents and caregivers of children 19 years of age and younger were eligible to participate in the survey. Membership in the ASD group was determined based on parent-reported primary diagnosis.

### Instrument

The survey was available in English, took ~10-min to complete, and consisted of 28 close-ended questions (Likert-type, matrix, single-answer, and multiple-answer) and one open-ended free-text question (Supplementary Methods 1). The survey questions were adapted from existing instruments (1, 23). To answer our research questions, the survey covered three key content areas: (1) technology use before and during the COVID-19 pandemic, (2) the parent perceived impact of technology use on parent perceived QoL and mental health, and (3) the diagnostic, sociodemographic, and family characteristics of respondents. Technology use was quantified as hours of use (in total, per device, and per activity) per day on weekdays and weekends (1). Parent's perception of the impact of technology use on child and family QoL and mental health was measured on a 5-point categorical scale from “very negative impact” to “very positive impact.” Lost time on other activities in favor of technology use and parents' emotions toward screen time (both measured on a 4-point categorical scale from “never” to “always”), as well as domains of benefit of technology use (e.g., coping with social isolation, online education, emotion regulation) were also surveyed. The diagnostic, sociodemographic, and family characteristics explored included household income and highest level of education, child's age, gender, race, diagnoses, and symptoms, if the child received educational support, the respondent's relationship to child, the number of children in the home, the number of adults responsible for childcare, the number of adults working inside and outside the home, and geographical information (first three digits of postal code). The survey concluded with a single open-ended free-text question, which provided a space for respondents to share anything else regarding their child's technology use during the COVID-19 school closures. For the purpose of the survey, “technology” referred to electronic screen media that included televisions, computers, laptops, game consoles, handheld consoles, virtual and augmented reality devices, smartphones, and tablets.

#### Community Involvement

The survey was developed in consultation with parents of children with ASD.

### Analyses

The data were analyzed in R 3.6.3 statistical programming (24). Outliers were identified and removed based on inspection of statistical distributions and measures of spread. We examined the significance of change during the COVID-19 period using a *t*-test with Bonferroni correction for two comparisons (weekend, weekday). Predictors of change were examined using multiple linear regression analysis, logistic regression, and ordinal logistic regression depending on the nature of the data. The primary predictors examined were group, age, and gender based on existing research on predictors of technology use (25–28). The results are reported with Bonferroni correction for three comparisons. Exploratory predictors examined were the number of adults working from home, number of siblings, household salary, and parental education, as well as the total screen time, the number of hours spent on different activities. These variables were chosen based on known predictors of screen time as well as demographics factors that may modulate the impact of COVID-19 on families (25, 29, 30). No multiple comparison correction was performed for the exploratory predictors. Regression diagnostics were used to evaluate the model fit with respect to heteroscedasticity and non-normality. Participants with incomplete data were included in the analyses where possible. Supplementary Table 1 provides the number of samples available for each of the demographic variables.

## Results

### Sample Characteristics

The sample consisted of 414 responses (ASD *n* = 127; community group *n* = 287). The community group was composed of children with no diagnosis (TD subgroup; *n* = 112) as well as those with diagnoses of attention deficit/hyperactivity disorder (ADHD; *n* = 17), anxiety disorder (*n* = 5), intellectual disability (*n* = 8), learning disability (*n* = 6), other (*n* = 10), and missing diagnosis information (*n* = 129). Seventy-one respondents were missing responses to our primary question and were removed from the analyses. The final sample consisted of 344 responses. The primary outcome (change in screen time) was not significantly different between the community group and those with missing diagnoses. Analyses were done with the entire community sample as well as the TD subgroup of the community sample (no missing diagnoses).

Table 1 details the demographic characteristics of these three groups. The majority of the participants were from the province of Ontario (89%), one of the Canadian provinces with the highest prevalence of COVID-19. As seen, both the community sample and its TD subgroup were significantly younger than the ASD group, had a larger proportion of females, and significantly higher household income and parental education, but not significantly different in race distribution or number of children in the household. To address the imbalance, two approaches were taken. First, the primary analyses were rerun with age, gender, household income, and parental education as covariates. Second, we subsampled the two groups to generate groups matched in age, gender, race distribution, number of children in the household, household salary, the number of adults responsible for childcare, working from home, and working outside the home (*n* = 156; Supplementary Table 2). The primary analyses were rerun on matched samples.

**Table 1:** Participant demographics.

	**ASD**	**Mixed community sample**	**TD subgroup**
**Age [mean (SD)]**	11.7 (4.06)	9.4 (4.26), *p* < 0.0001	8.4 (4.12), *p* < 0.0001
**Gender (Male:Female)**	28:99	65:96, *p* = 0.001	47:65, *p* = 0.001
**Race**		*p* = 0.16	*p* = 0.10
Black	4%	2%	3%
East Asian	6%	10%	12%
First Nations/Metis/Inuit	4%	1%	1%
Latin American	6%	1%	2%
Middle Eastern	4%	2%	2%
South Asian	3%	5%	4%
Southeast Asian	4%	4%	5%
White	63%	67%	66%
Other	2%	3%	4%
Prefer not to say	4%	3%	1%
**Annual household income**		*p* < 0.0001	*p* < 0.0001
< $29,999	12%	1%	1%
$30,000–49,999	9%	6%	4%
$50,000–74,999	16%	5%	5%
$75,000–99,999	13%	9%	8%
> $100,000	37%	69%	64%
Prefer not to say	13%	11%	10%
**Education**		*p* = 0.0002	*p* < 0.0001
Secondary school	1%	2%	1%
Elementary school	1%	0%	0%
College	25%	8%	4%
University	69%	83%	87%
Other	2%	7%	6%
Prefer not to say	2%	1%	2%

*P-values are reported for comparisons relative to the ASD group. ASD, autism spectrum disorder; TD, typically developing; SD, standard deviation*.

The majority of participants in the ASD group communicated verbally (83%). They either attended regular classrooms (26%), received support in a regular classroom (30%), or attended a special education classroom (32%). The most frequently co-occurring symptoms or conditions in this group were anxiety disorders (34%), ADHD (31%), and learning disability (26%). Supplementary Table 3 provides detailed characteristics of the group. For both the ASD and community groups, the majority of respondents were mothers (ASD: 91%, community: 88%), followed by fathers (ASD: 8%, community: 10%). The number of adults responsible for childcare [ASD: mean = 1.9 (SD = 0.56), community: mean = 2.2 (SD = 0.57), *p* < 0.0001] and number of adults working from home [ASD: mean = 0.8 (SD = 0.71), community: mean = 1.2 (SD = 0.71), *p* < 0.0001] were both significantly higher in the community group. The number of adults working outside the home at least 1 day a week was not significantly different between the groups.

### Screen Time

Pre-pandemic, mean total screen time in the ASD group was perceived by parents to be 3.3 h [95% confidence interval (CI) 2.92, 3.63] on weekdays and 4.9 h (95% CI 4.55, 5.27) on weekends. In the community sample, mean pre-pandemic screen time was perceived as 2.0 h (95% CI 1.71, 2.34) on weekdays and 3.7 h (3.34, 3.98) on weekends. During the COVID-19 pandemic school closures, mean weekday and weekend total screen time during for the ASD group was 6.9 h (95% CI 6.49, 7.21) and 6.3 h (95% CI 5.91, 6.63) of use, respectively, while the community sample reported 5.6 h (95% CI 5.28, 5.92) and 5.0 h (95% CI 4.70, 5.34) of use, respectively. Compared to the community sample, the ASD group had significantly higher screen time use before and during the pandemic on weekdays and weekends [weekdays: difference = 1.14 (SE = 0.18), *t* = 6.56, *p* < 0.0001; weekends: difference = 1.41 (SE = 0.20), *t* = 6.93, *p* < 0.0001]. The conclusions did not change when controlling for age, gender, parental education, and income, or after rerunning the analyses with the TD and matched subgroups.

**Figure 1:**
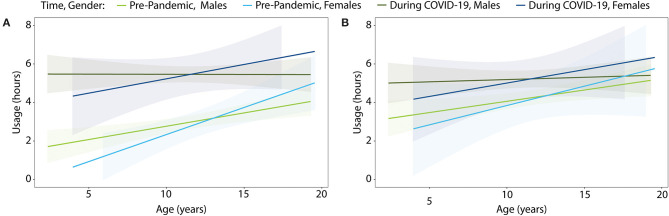
Screen time usage by age for children with ASD before and during the COVID-19 school closures on **(A)** weekdays and **(B)** weekends. Colored shading denotes 95% confidence interval.

Analyses revealed a significant increase in total screen time during the pandemic (vs. before) in the ASD group (Figure 1; two-sided *t*-test; weekdays: mean difference = 3.8 h [95% confidence interval (CI) 3.35, 4.25], *p* < 0.0001; weekends: mean difference = 1.5 h [95% CI 28 1.17, 1.92], *p* < 0.0001). For the ASD group, there was no significant effect of age or gender on the change in screen time.

**Figure 2:**
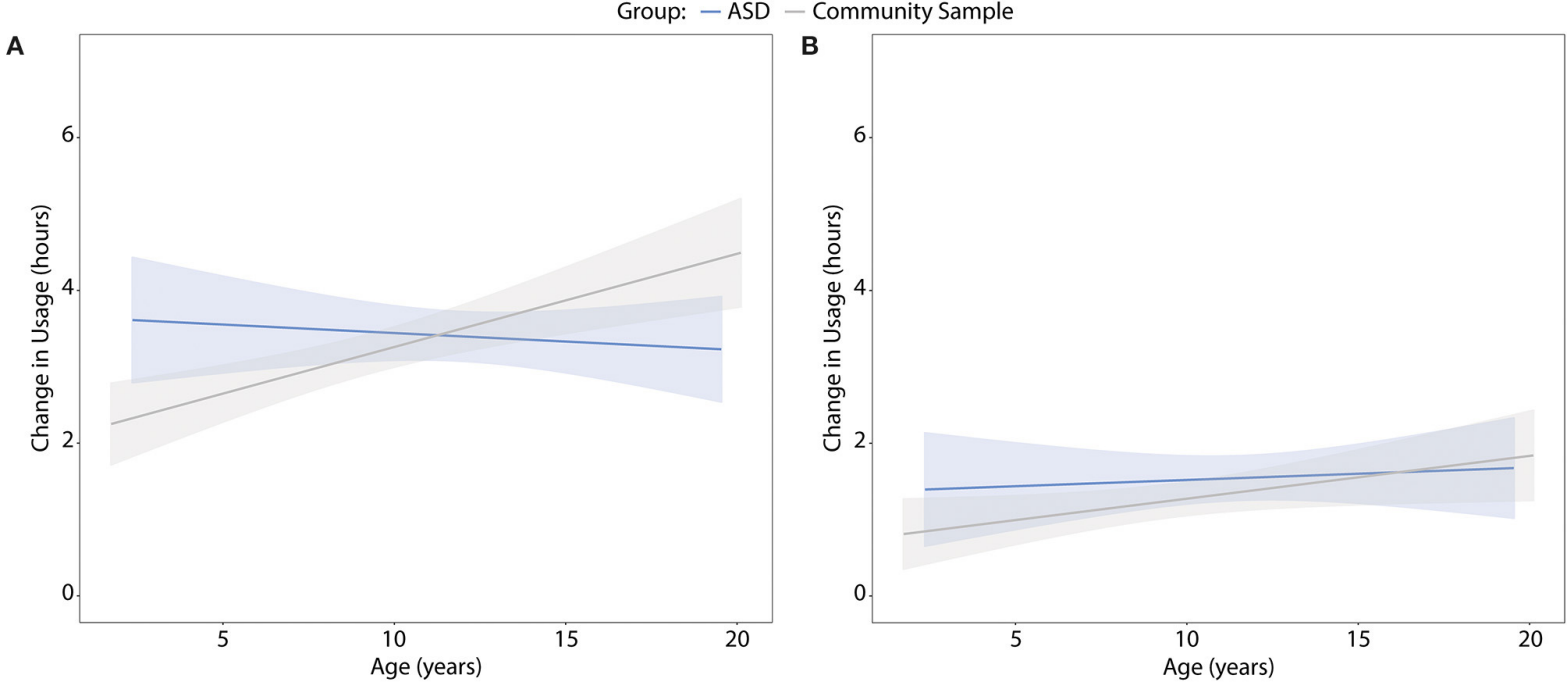
Change in screen time by age during the COVID-19 school closures for children with ASD and the community sample on **(A)** weekdays and **(B)** weekends. Colored shading denotes 95% confidence interval.

When looking at the entire sample, there was a significant age by group interaction, suggesting increased change in screen time for younger children with ASD relative to the community sample {Figure 2; weekdays: difference in regression coefficients = 0.2 [standard error (SE) = 0.06], *p* = 0.001; weekends: difference = 0.1 (SE = 0.05), *p* = 0.019}. The interaction remained significant when the analyses were rerun with the TD subgroup (weekdays only), but not with the matched sample. The latter was likely due to limited age range in the matched sample.

**Figure 3:**
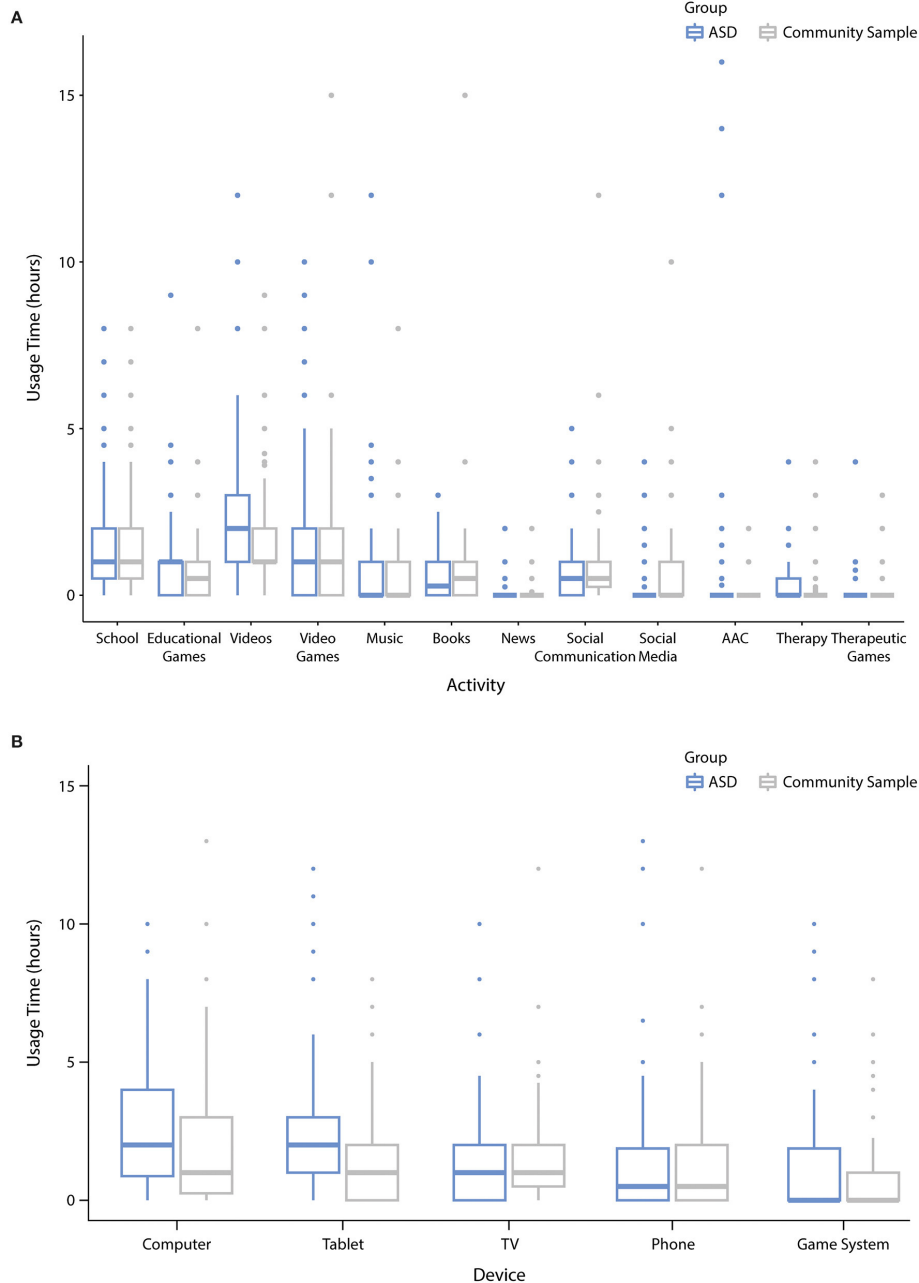
Distribution of screen time during the COVID-19 closures across different **(A)** activities and **(B)** devices. Center line represents the median (50% quartile), box limits represent upper (75%) and lower (25%) quartiles, whiskers represent 1.58× interquartile range (IQR). Outliers are represented as data points beyond the end of the whiskers.

In terms of the distribution of screen time (Figure 3A), the longest screen time durations were reported for watching videos [ASD: mean hours = 2.4 (SD = 2.09); community: mean hours = 1.7 (SD = 1.47)], playing video games [ASD: mean hours = 1.6 (SD = 2.18); community: mean hours = 1.2 (SD = 1.94)], and engaging in online learning [ASD: mean hours = 1.6 (SD = 1.63); community: mean hours = 1.7 (SD = 1.68)]. The highest usage (Figure 3B) was reported for tablets [ASD: mean hours = 2.7 (SD = 2.93); community: mean hours = 1.5 (SD = 1.65)] and computers/laptops [ASD: mean hours = 2.4 (SD = 2.31); community: mean hours = 2.1 (SD = 2.22)].

### Parent Perceived Impact

**Figure 4:**
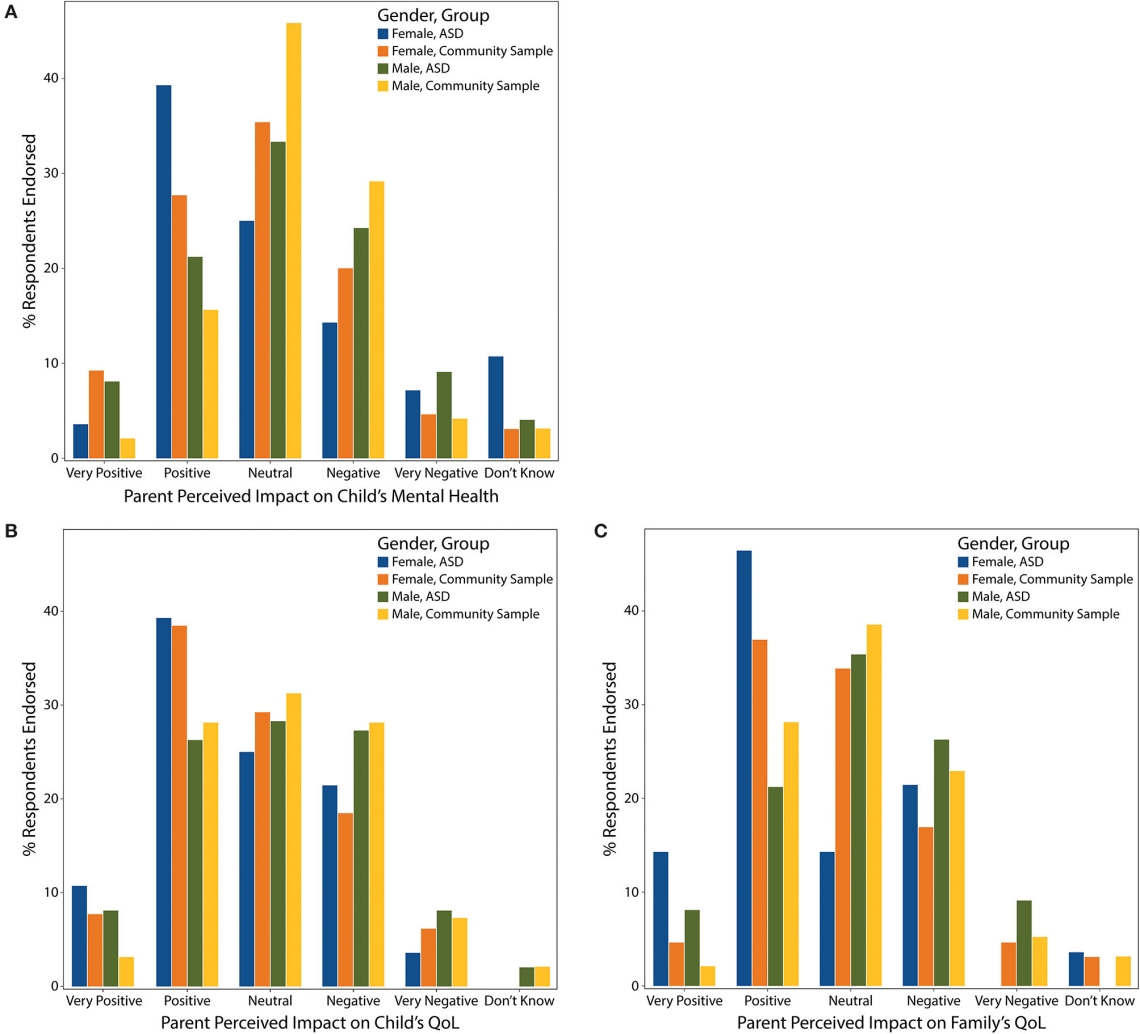
Perceived impact of screen time on parent perceived **(A)** child's mental health, **(B)** child's QoL, and **(C)** family's QoL displayed by gender and group.

Our results revealed mixed perceptions of impact of screen time on parent perceived child and family QoL and mental health, with nearly equal proportions of respondents endorsing positive and negative impact (Figure 4). There was no significant effect of group or age, but gender was a significant predictor of both parent perceived QoL and mental health, with male gender associated with a higher likelihood of a negative perceived impact [QoL (child/family) odds ratio (OR) = 1.8 (95% CI 1.1, 2.9), corrected *p* = 0.040; mental health OR = 1.9 (95% CI 1.1,3.1), corrected *p* = 0.0028]. The conclusions remained unchanged when the analyses were rerun with the TD and matched subgroups.

Supplementary Table 4 provides the results of ordinal regression for the exploratory predictors. Higher likelihood of negative perceived impact was associated with increased screen time on weekdays [family QoL OR = 1.1 (95% CI 1.02, 1.23), *p* = 0.02; child's mental health OR = 1.1 (95% CI 1.04, 1.25), *p* = 0.006] and weekends [family QoL OR = 1.1 (95% CI 1.01, 1.21), *p* = 0.04], and the number of hours playing video games [family QoL OR = 1.1 (95% CI 1.00, 1.26), *p* = 0.04] and watching videos [family QoL OR = 1.1 (95% CI 1.02, 1.28), *p* = 0.02] on weekends. Lower likelihood of negative perceived impact was associated with screen time spent connecting with friends and family on weekdays [child's QoL OR = 0.7 (95% CI 0.56, 0.92), *p* = 0.01; family QoL OR = 0.8 (95% CI 0.61, 1.0), *p* = 0.05; child's mental health OR = 0.7 (95% CI 0.53, 0.87), *p* = 0.002], playing educational games on weekends [child's QoL OR = 0.7 (95% CI 0.53, 1.012), *p* = 0.05], using therapeutic apps on weekdays [child's QoL OR = 0.5 (95% CI 0.28, 0.99), *p* = 0.05], and weekday time spent on social media [family QoL OR = 0.7 (95% CI 0.50, 1.01), *p* = 0.05; child's mental health OR = 0.6 (95% CI 0.44, 0.89), *p* = 0.008].

**Figure 5:**
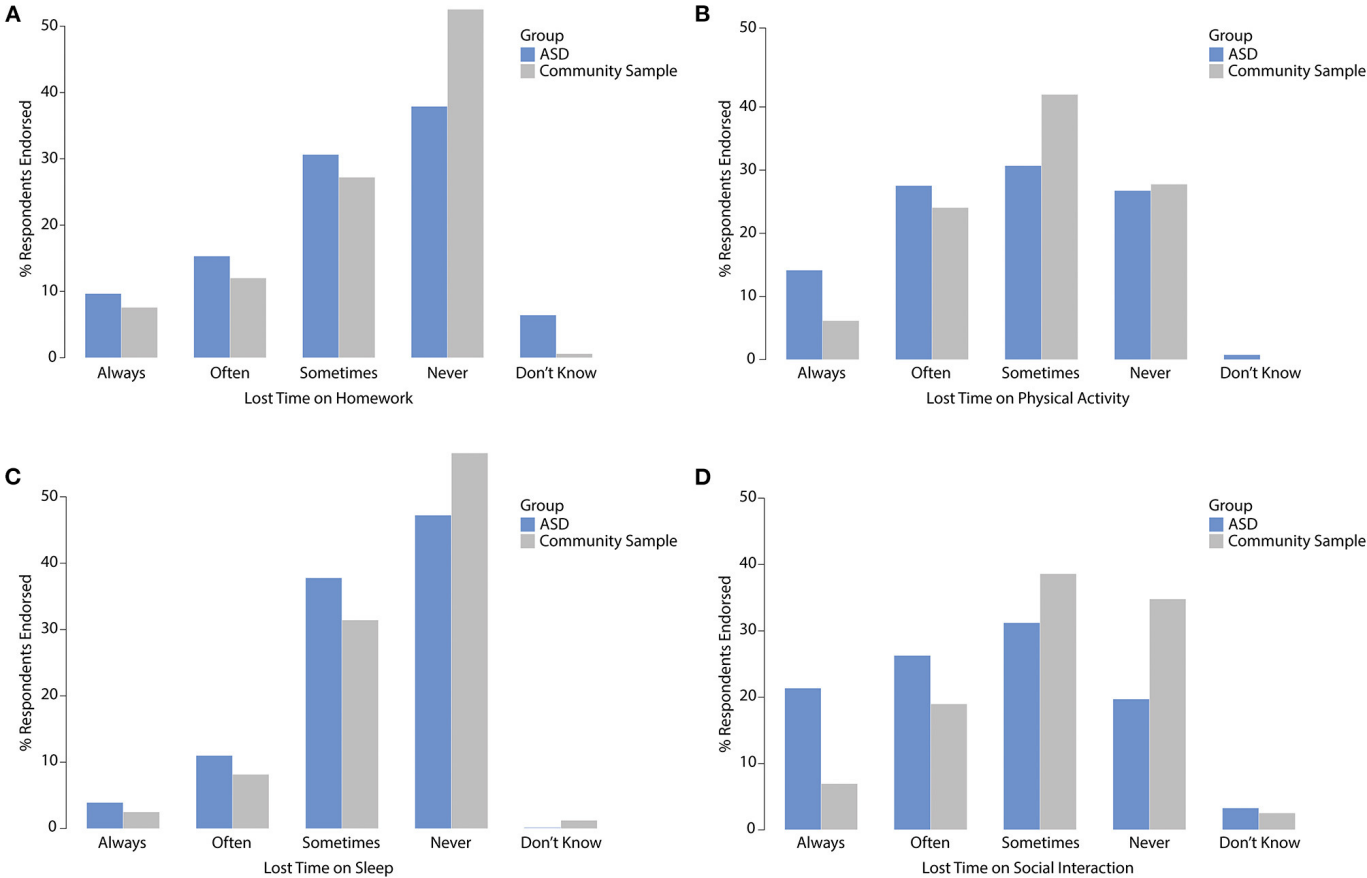
Children's lost time on **(A)** homework, **(B)** physical activity, **(C)** sleep, and **(D)** social interactions due to screen time.

To further probe the specific domains of negative perceived impact, the respondents were asked to indicate how often children lost time on sleep, homework, physical activity, or social interactions because of screen time (Figure 5). For the ASD group, always/often choices were most frequently endorsed for losing time on social interactions (47%) and physical activity (41%). Ordinal logistic regression revealed significantly more lost time on social interactions for the ASD group compared to the community sample [OR = 2.7 (95% CI 1.66, 4.36), *p* < 0.0001]. Marginal group effects were also found for lost time on homework [OR = 1.6 (95% CI 0.97, 2.65), *p* = 0.06] and physical activity [OR = 1.5 (95% CI 0.96, 2.47), *p* = 0.07]. The effects of age and gender were not significant.

**Figure 6:**
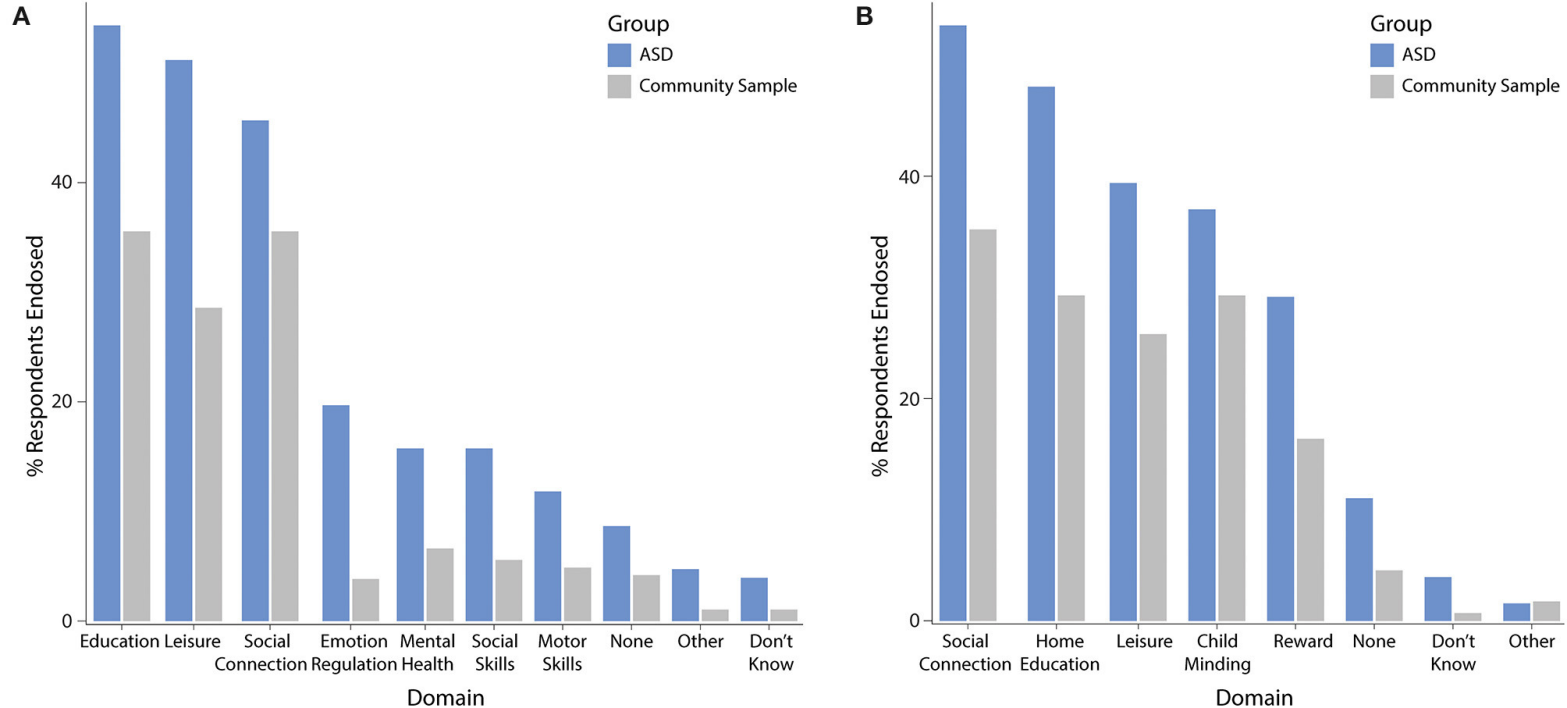
Perceived screen time benefits for **(A)** the child and **(B)** the family.

The most frequently endorsed domains of benefit for children (Figure 6A) were online education (ASD: 54%, community sample: 36%), leisure (ASD: 51%, community sample: 29%), and coping with social isolation (ASD: 46%, community sample: 36%). Nine percent (ASD) and 4% (community sample) of parents endorsed no benefits of screen time for the child. The most frequently endorsed benefit domains for the family (Figure 6B) were similar and included coping with social isolation (ASD: 53%, community sample: 35%), home schooling (ASD: 48%, community sample: 29%), leisure (ASD: 39%, community sample: 26%), followed by child minding (ASD: 37%, community sample: 29%), and using technology as reward (ASD: 29%, community sample: 16%). No benefits of screen time for the family were endorsed by 11 and 5% of the ASD group and community sample, respectively.

For the child, the community sample had a higher likelihood of benefiting from screen time to cope with social isolation [OR = 2.4 (95% CI 1.43, 4.1), corrected *p* = 0.003], but decreased likelihood of benefiting in the emotion regulation domain [OR = 0.27 (95% CI 0.11, 0.59), corrected *p* = 0.003]. Age was positively associated with benefits in social coping [OR = 1.1 (95% CI 1.02, 1.15), corrected *p* = 0.03]. For the family, age was negatively associated with using technology as reward [OR = 0.86 (95% CI 0.81, 0.93), corrected *p* < 0.0001] and child minding [OR = 0.9 (95% CI 0.82, 0.93), corrected *p* < 0.0001].

### Parental Emotions Toward Screen Time

The most frequently endorsed emotions by parents were (endorsed as always or often; Supplementary Figure 1): guilt (ASD: 48%; community: 53%), frustration (ASD: 40%; community: 32%), worry (ASD: 39%; community: 36%), and relieved (ASD: 37%; community: 27%). The least frequently endorsed emotions were excitement (ASD: 12%; community: 8%), anger (ASD: 15%; community: 13%), and hope (ASD: 19%; community: 10%). Examining the predictors of the top five emotions did not reveal a group effect but did show an increased likelihood of negative feelings with increasing screen time (Supplementary Table 5), and a negative association of age with feelings of guilt [OR = 1.1 (95% CI 1.02, 1.14), *p* = 0.006].

## Discussion

Our results showed a significant increase in screen time for children and youth with ASD during the COVID-19 pandemic (3.8 h on weekdays, 1.5 h on weekends). This is in line with other studies of technology use during COVID-19 in the general pediatric population across the world (1, 2, 4). For example, in their longitudinal survey study in Shanghai, China, Xiang et al. (4) found that screen time of children and adolescents surged by ~30 h per week from January to March 2020. Another pre-peri lockdown longitudinal study conducted in Italy (2), found that leisure screen time of children and adolescents increased by more than 4.5 h per day. These studies took place in countries that were experienced early COVID-19 outbreaks (Italy, China) (31). How and to what extent pandemic measures were enforced (e.g., stricter lockdowns), the density of living arrangements (e.g., multi-family residential housing), and the built environment (e.g., availability of parks and outdoor spaces) may be contributors to the observed variability in screen time increases. Furthermore, seasonal differences in behavior may have contributed to screen time behaviors for both learning and leisure, as the present study was conducted at the end of the school year in spring/summer as opposed to late winter in previous studies. Regarding total weekday and weekend screen time during the COVID-19 school closures, the ASD group exhibited 6.9 and 6.3 h of use, respectively, while the community sample reported 5.6 and 5.0 h of use, respectively. The latter is consistent with the results of another pandemic survey study of a representative, nation-wide sample of Canadian children (*n* = 1,472), in which average daily screen time was calculated at 5.1 h for children (5–11 years) and 6.5 h for youth (12–17 years) (1). Collectively, these findings suggest that, as a salient consequence of the pandemic response measures, children and youth are spending a substantial proportion of their days engaged in screen time. This raises concerns over the long-term and developmental ramifications of such extensive use. At the same time, it is important to note that current screen time recommendations (32) are based on pre-pandemic evidence and specified solely for leisure use. Given the unprecedented circumstances of the COVID-19 pandemic, and the novel context of technology use, there is a critical need for revision of screen time recommendations to reflect the current needs of children and families.

We anticipated that children with ASD would exhibit greater surges in screen time during school closures compared to other children due to loss of supports and services, potential use of screen time by families to manage challenging behaviors, and difficulties arising from intense and restricted pre-occupations related to screen use. Interestingly, our results did not confirm this. This may be due to the elevated pre-pandemic screen time levels observed in this population, or a result of a marked loss of opportunities for in-person participation in social and leisure activities for the community sample, as these opportunities are often limited for children with ASD even without the pandemic measures in place.

We also did not find an association between change in screen time and other demographic and socio-economic factors. This was unexpected given the differential association of technology use with gender, race, and socioeconomic status (25, 27–29), and the many inequities moderating the impact of COVID-19 on families (33, 34). Given our limited number of responses from racialized and lower income families, further research targeted at marginalized populations is needed.

Compared to the ASD group, there was a significantly steeper increase in change in screen time with age for the community sample. A positive association of age and screen time is well-documented in the literature (35–37), however, there is some research to suggest a milder age gradient for children with ASD (38). Similarly, results of the present study suggest children with ASD have exhibited a more consistent change in screen time across the age span during COVID-19, resulting in an increased change in screen time for younger children with ASD compared to the community sample. This difference may be related to younger children with ASD relying more heavily on screens for entertainment or distraction than other children during the COVID-19 school closures, or the more significant loss of school-based supports. These results may reflect a greater dependence on screen time to cope with social isolation in adolescents in the community sample, but not those with ASD. Further research is needed to validate this.

The impact of screen time on children and families during the COVID-19 pandemic reported by parents was mixed regarding perceived mental health and QoL, with male gender associated with higher likelihood of negative impact despite a lack of significant differences in screen time or increase in use compared to females. This concern is supported by research detailing greater adverse effects of screen time for males in terms of specific mental health [e.g., pathological gaming (39)] and physical health domains [e.g., obesity (40), adiposity (41), and metabolic risk factors (42)], though reported effects are mixed (43, 44). The differences observed in this study may also be a reflection of parental attitudes toward specific screen time behaviors (e.g., video gaming compared to social media use), how parents experience screen time behaviors of their children (e.g., video game play may be more disruptive), or a difference in how boys and girls are spending time on each device or activity (e.g., violent vs. non-violent video games, types of videos watched). The likelihood of negative perceived impact was also found to be positively associated with total screen time, number of hours playing video games and time spent watching videos, but negatively associated with time spent connecting with friends and family, on social media, playing educational games, and using therapeutic apps. Interestingly, although online learning accounted for a large proportion of screen time, the amount of time spent online learning was not predictive of the parent perceived impacts of screen time during the COVID-19 school closures. Once replicated in other samples, these findings can lead to formulation of more activity-specific recommendations to optimize the impact of screen time on mental health and QoL.

The ASD group and the community sample reported similar benefits of screen time during the COVID-19 pandemic for their children's social connectivity, education, and leisure time; however, the community sample had a higher likelihood of benefitting from screen time to cope with social isolation. Conversely, almost half of children with ASD frequently lost time on social interactions due to their technology use during the COVID-19 pandemic, a rate significantly higher than for the community sample. Due to the hallmark characteristics of ASD (17), it may be that children with ASD have greater difficulty engaging in social interactions given the physical and social distancing requirements of the pandemic response, or have a smaller pool of peers with whom they could socialize (45). Alternatively, children with ASD may exhibit a preference for non-social screen time activities as these may be less emotionally and cognitively demanding. Parents of children with ASD further reported disproportionately affected physical activity and time spent on homework due to screen time. Despite similar increases in screen time during the pandemic period, children with ASD appear to be at increased risk for these negative outcomes. This may be due to their higher overall screen time compared to other children or may be related to characteristic traits of the disorder (i.e., restricted or intense interests) that change how children with ASD engage during screen time. Our findings reflect the proposed consequences of technology use in children with ASD (15), and raises concerns over compounding negative outcomes for this population in instances of prolonged school closures.

Parents' emotions toward screen time were overwhelmingly negative with guilt, frustration, and worry as the most frequently endorsed emotions, a finding that transcended diagnoses, socio-economic, and demographic characteristics (except age). Unsurprisingly, these negative emotions were predicted by total screen time in our exploratory analyses, and feelings of guilt in particular were negatively associated with age. However, parental concerns over screen time are not unique to the pandemic period; prior research indicates as many as 84% of parents report concerns over their children's screen use (46). The feeling of relief was also frequently endorsed by parents and may be attributed to the role digital devices have played in educating, regulating emotion, and child minding (i.e., screen time babysitting). While there are fewer alternatives to screen time for kids during the COVID-19 school closures, parental mental health needs specially related to feelings of guilt over the amount screen time may be actionable targets.

### Strengths

This study has several strengths and represents a significant and timely contribution to the literature. To our knowledge, this is the first study to investigate changes in screen time in children and youth with ASD and assess how that change compares to other children in Canada. A recent nation-wide parent-report survey conducted by ParticipACTION assessed movement behaviors of Canadian children and youth, including sedentary screen time, ~1-month after COVID-19 was declared a global pandemic (1). The present study extends this research by quantifying the change in screen time, examining these changes following more prolonged exposure to the pandemic response (2- to 3-months), and investigating screen time in a clinical population of youth. This study is also the first to investigate the parent perceived impact of increased screen time on children and families' mental health and quality of life during the COVID-19 pandemic in Canada. The results of the exploratory analyses, once replicated, can yield actionable targets that can be used to inform guidelines and recommendations for families, and support specific activities for optimal engagement with technology for children and youth during the COVID-19 school closures. Specifically, screen time appears to be more beneficial for the child and family when it is used as a means to socially connect with others, to learn new skills, and to support one's mental health through therapeutic apps.

### Limitations

The results of this must be interpreted in the context of several limitations. First, all outcomes were based on parent-report rather than direct quantitative measurement and may be subject to response bias. Parent's awareness of their children's screen time behavior may be facilitated or hindered by their own routine changes due to the COVID-19 pandemic, such as working from home, increased childcare demands, and financial and psychological stressors. Moreover, parents who are not actively engaged in screen time with their children may not be able to accurately account for their technology use. Secondly, as parents were not required to answer every question to complete the survey, there is uncertainty over those left unanswered (e.g., missing diagnosis information). To address this, we showed that the conclusions remained similar when the analyses were rerun on subgroups and matched samples where appropriate. A third limitation is that our sample included a disproportionately low number of families with lower household incomes (47). Despite this limitation, the observed screen time in the community sample during the COVID-19 school closures was in line with that of a recent representative, nation-wide sample of screen time in Canadian children during the pandemic period (1). Lastly, due to our survey distribution strategy, we were unable to calculate the response rate. It is possible that non-respondents differ from respondents in ways that are pertinent to the aims of the survey [non-response effect (48)].

## Conclusions

The present survey characterizes an increase in screen time during the COVID-19 school closures in the Canadian pediatric ASD population, with the largest increases in weekday use. Although children with and without ASD exhibited comparable screen time increases, children with ASD were found to have significantly greater usage both prior to and during the pandemic. Parents' perceived impact of screen time on child and family mental health and quality of life during the pandemic was mixed, though a stronger negative influence for males was found. Children were found to benefit most from screen time for social connections, education, and leisure time; however, children with ASD were less likely to benefit from screen time to cope with social isolation than other children. In fact, screen time of children with ASD resulted in significantly more lost time on social interactions compared to the community sample, which may exacerbate difficulties in this area and raises concerns over compounding negative outcomes for this population from prolonged school closures. Parental feelings toward their children's screen time use were overwhelmingly negative, with guilt, frustration, and worry as the most frequently endorsed emotions. Given the unprecedented circumstances of the COVID-19 pandemic, and the novel context of technology use during the pandemic school closures, the findings of this study highlight the need for revision of screen time recommendations to reflect the current needs of children and families.

## Data Availability Statement

The datasets presented in this article are not readily available because public disclosure of data was not included in the research ethics approval or participant informed consent.

## Ethics Statement

The studies involving human participants were reviewed and approved by Research Ethics Board of Holland Bloorview Kids Rehabilitation Hospital. The participants provided electronic informed consent to participate in this study.

## Author Contributions

AK conceptualized the study, designed the study, created the data collection instrument, developed the data analysis plan, performed the data analyses, created the figures, interpreted the data, and critically reviewed and revised the manuscript. RC designed the study, created the data collection instrument, managed survey distribution, created the figures, interpreted the data, drafted the initial manuscript, and critically reviewed and revised the manuscript. JZ performed the initial literature review, created the data collection instrument, and managed survey distribution. AD developed the data analysis plan, interpreted the data, and critically reviewed and revised the manuscript for important intellectual content. EA, EB, SM, JB, and MP contributed to conceptualizing the study, interpreted the data, and critically reviewed and revised the manuscript. All authors approved the final manuscript and agree to be accountable for all aspects of the work.

## Conflict of Interest

AK reports personal fees from Shaftesbury outside the submitted work. In addition, AK has a patent US 9,844,332 B2 with royalties paid to Awake Labs and a patent US 16/276,208 pending to Awake Labs. AK is the inventor of a software called the Anxiety Meter. She is involved in commercializing the Anxiety Meter and will financially benefit from its sales. AK served on the board of advisors for Shaftesbury, a media company developing virtual reality products for children with ASD, from February 2020–February 2021, and was compensated financially for this role. SM receives royalties from Springer for her book, Assessing and Treating Anxiety Disorders in Young Children. EA reports grants from Roche, personal fees from Roche, personal fees from Quadrant, personal fees from Wiley, book royalties from Springer, book royalties from APPI, and non-financial support from AMO Pharma outside the submitted work. In addition, EA has a patent US 9,844,332 B2 with royalties paid to Awake Labs. EA holds a patent on the software called the Anxiety Meter; the Anxiety Meter is being commercialized and she will financially benefit from its sales. The remaining authors declare that the research was conducted in the absence of any commercial or financial relationships that could be construed as a potential conflict of interest.

## Publisher's Note

All claims expressed in this article are solely those of the authors and do not necessarily represent those of their affiliated organizations, or those of the publisher, the editors and the reviewers. Any product that may be evaluated in this article, or claim that may be made by its manufacturer, is not guaranteed or endorsed by the publisher.
