# Supplementary material for: Characterizing Changes in Screen Time During the COVID-19 Pandemic School Closures in Canada and Its Perceived Impact on Children With Autism Spectrum Disorder
Source: Front Psychiatry. 2021 Aug 18;12:702774. doi: 10.3389/fpsyt.2021.702774 (PMC8416261; doi:10.3389/fpsyt.2021.702774)
Supplement: Supplementary file 1 [file Data_Sheet_1.docx]

#### **Supplementary Material**

**Supplementary Table 1.** Available sample size (N) per group for each of the demographic variables used in the analyses.

**Supplementary Table 2.** Characteristics of the matched groups.

**Supplementary Table 3.** Characteristics of the ASD and community sample groups.

**Supplementary Table 4.** Predictors of impact.

**Supplementary Table 5.** Predictors of parental emotions.

**Supplementary Figure 1.** Parental emotions associated with children’s screen time for the ASD and community sample groups.

**Supplementary Methods 1.** Survey instrument.

####

#### **Supplementary Table 1.** Available sample size (N) per group for each of the demographic variables used in the analyses. ASD: Autism spectrum disorder, TD: Typically developing

| **Variable** | **N (ASD)** | **N (Mixed Community Sample)** | **N (TD Subgroup)** |
| --- | --- | --- | --- |
| Change in weekday screen time | 126 | 218 | 110 |
| Change in weekend screen time | 126 | 217 | 110 |
| Gender | 127 | 161 | 112 |
| Age | 121 | 153 | 106 |
| Parent education | 126 | 160 | 112 |
| Household income | 126 | 160 | 112 |
| Number of children in the household | 126 | 161 | 112 |
| Number of adults responsible for childcare | 122 | 158 | 110 |
| Number adults working outside the home | 125 | 158 | 111 |
| Number adults working from home | 126 | 160 | 112 |

####

#### **Supplementary Table 2.** Characteristics of the matched groups. ASD: Autism spectrum disorder, SD: Standard deviation.

| **Variable** | **ASD (n=78)** | **Matched Community Sample (n=78)** | **P Value**  **(Group Effect)** |
| --- | --- | --- | --- |
| **Age (years), mean (SD)** | 10.9 (3.96) | 10.7 (3.70) | 0.79 |
| **Gender (Male:Female)** | 63:15 | 63:15 | 1.00 |
| **Number adults responsible for childcare, mean (SD)** | 2.0 (0.43) | 2.0 (0.43) | 1.00 |
| **Number adults working from home, mean (SD)** | 1.0 (0.69) | 1.0 (0.69) | 1.00 |
| **Number adults working outside the home, mean (SD)** | 0.6 (0.61) | 0.6 (0.59) | 0.59 |
| **Number children in the household, mean (SD)** | 2.0 (0.87) | 2.1 (0.84) | 0.50 |
| **Race** |  |  | 0.50 |
| Black | 1.1% | 1.2% |  |
| East Asian | 3.5% | 4.9% |  |
| First Nations/Metis/Inuit | 3.5% | 2.5% |  |
| Latin American | 5.8% | 0.0% |  |
| Middle Eastern | 5.8% | 2.4% |  |
| South Asian | 2.3% | 4.9% |  |
| Southeast Asian | 1.1% | 2.4% |  |
| White | 70.1% | 72.8% |  |
| Other | 2.3% | 2.4% |  |
| Prefer not to say | 3.5% | 6.1% |  |
| **Annual household income** |  |  | 0.85 |
| < $29,000 | 1.3% | 1.3% |  |
| $30,000 - $49,999 | 7.7% | 6.4% |  |
| $50,000 - $74,999 | 10.3% | 6.4% |  |
| $75,000 - $99,999 | 15.4% | 11.5% |  |
| > $100,000 | 50.0% | 60.2% |  |
| Prefer not to say | 15.4% | 14.1% |  |
| **Education** |  |  | 0.19 |
| Secondary school | 0% | 2.6% |  |
| Elementary school | 1.3% | 0% |  |
| College | 17.9% | 9.0% |  |
| University | 76.9% | 84.6% |  |
| Other | 2.6% | 3.8% |  |
| Prefer not to say | 1.3% | 0% |  |

**Supplementary Table 3.** Characteristics of the ASD and community sample groups. ASD: Autism spectrum disorder, TD: Typically developing.

|  | **ASD** | **Community Sample** | **TD Subgroup** |
| --- | --- | --- | --- |
| **School supports** |  |  |  |
| Regular classroom | 26% | 62% | 73% |
| Receive support in regular classroom | 30% | 15% | 6% |
| Special education classroom | 32% | 5% | 0% |
| Gifted program | 2% | 2% | 3% |
| Do not attend school | 1% | 6% | 9% |
| Other | 8% | 7% | 7% |
| Missing | 0% | 45% | 2% |
| **Co-occurring conditions/symptoms** |  |  |  |
| ASD | - | 2% | 0% |
| Attention deficit/hyperactivity | 31% | 4% | 3% |
| Anxiety | 34% | 7% | 4% |
| Depression | 2% | 2% | 1% |
| Intellectual disability | 17% | 2% | 0% |
| Learning disability | 26% | 4% | 0% |
| Obsessive compulsive disorder | 9% | 1% | 2% |
| Attention difficulties | 18% | 8% | 6% |
| Sleep difficulties | 23% | 8% | 10% |
| None | 10% | 32% | 73% |
| Other | 8% | 3% | 1% |
| Missing | 9% | 48% | 4% |
| **Mode of communication** |  |  |  |
| Does not communicate verbally | 10% | 1% | 0 |
| Uses augmented and alternative communication | 11% | 1% | 0 |
| Uses sign language | 1% | 1% | 1% |
| Writes on a computer | 5% | 1% | 3% |
| Doesn’t have words but uses non-verbal cues | 8% | 2% | 1% |
| Uses verbal communication (speaks words and sentences) | 83% | 53% | 94% |
| Other | 3% | 1% | 2% |
| Missing | 0% | 45% | 2% |

#### **Supplementary Table 4.** Predictors of impact. The odds-ratios indicate the likelihood of negative impact. Reported as odds-ratio (95% confidence interval). Reported p-values are uncorrected. QoL: Quality of life, n.s.: Not significant, AAC: Augmentative and alternative communication.

| **Predictor** | **Child QoL** | **Family QoL** | **Mental Health** |
| --- | --- | --- | --- |
| **Group** | n.s. | n.s. | n.s. |
| **Age** | n.s. | n.s. | n.s. |
| **Gender** | 1.8 (1.13, 2.98), p=0.014 | 1.9 (1.14, 3.0), p=0.012 | 1.9 (1.18, 3.19), p=0.009 |
| **Total screen time (weekdays)** | 1.1 (1.00, 1.21), p=0.051 | 1.1 (1.02, 1.23), p=0.020 | 1.1 (1.04, 1.25), p=0.006 |
| **Total screen time (weekends)** | n.s. | 1.1 (1.01, 1.21), p=0.036 | n.s. |
| **Total number of adults** | n.s. | n.s. | n.s. |
| **Number of adults working from home** | n.s. | n.s. | n.s. |
| **Number of adults working outside** | n.s. | n.s. | n.s. |
| **Number of children in the household** | n.s. | n.s. | n.s. |
| **Household salary** | n.s. | n.s. | n.s. |
| **Parent education** | n.s. | n.s. | n.s. |
| **Type of screen time activity** |  |  |  |
| AAC | 0.8 (0.66, 0.99), p=0.04 ^†^ | 0.8 (0.66, 0.98), p=0.03 ^†^ | n.s. |
| Connecting with friends/family | 0.7 (0.56, 0.92), p=0.01 ^†^ | 0.8 (0.61, 1.0), p=0.05 ^†^ | 0.68 (0.53, 0.87), p=0.002 ^†^ |
| Connecting with therapists | n.s. | n.s. | n.s. |
| Playing educational games | 0.7 (0.53, 1.012), p=0.05 ^§^ | n.s. | n.s. |
| Listening to music | n.s. | n.s. | n.s. |
| Reading books | n.s. | n.s. | n.s. |
| Reading the news | n.s. | n.s. | n.s. |
| School work/online learning | n.s. | n.s. | n.s. |
| Social media | n.s. | 0.7 (0.50, 1.01), p=0.05 ^†^ | 0.62 (0.44, 0.89), p=0.008 ^†^ |
| Playing therapeutic apps | 0.5 (0.28, 0.99), p=0.05 ^†^ | n.s. | n.s. |
| Playing video games | n.s. | 1.1 (1.00, 1.26), p=0.04 ^§^ | n.s. |
| Watching videos | n.s. | 1.1 (1.02, 1.28), p=0.02 ^§^ | n.s. |
| ^†^ Weekday use  ^§^ Weekend use | | | |

####

#### **Supplementary Table 5.** Predictors of parental emotions. The odds-ratios indicate the likelihood of negative impact. Reported as odds-ratio (95% confidence interval). Reported p-values are uncorrected. N.s.: Not significant, AAC: Augmentative and alternative communication,

| **Predictor** | **Guilt** | **Frustration** | **Worry** | **Relief** |
| --- | --- | --- | --- | --- |
| **Group** | n.s. | n.s. | n.s. | n.s. |
| **Age** | 1.1 (1.02, 1.14),  p=0.006 | n.s. | n.s. | n.s. |
| **Gender** | n.s. | n.s. | n.s. | n.s. |
| **Total weekday screen time** | 0.9 (0.78, 0.95),  p=0.002 | 0.9 (0.80, 0.97),  p=0.008 | 0.85 (0.77, 0.93),  p=0.0008 | n.s. |
| **Total weekend screen time** | 0.8 (0.76, 0.91),  p<0.0001 | 0.8 (0.76, 0.92),  p=0.0002 | 0.8 (0.75, 0.91),  p<0.0001 | n.s. |
| **Total number of adults** | n.s. | n.s. | n.s. | n.s. |
| **Number of adults working from home** | n.s. | n.s. | n.s. | n.s. |
| **Number of adults working outside** | n.s. | n.s. | n.s. | n.s. |
| **Number of children in the household** | n.s. | n.s. | n.s. | n.s. |
| **Household salary** | n.s. | n.s. | n.s. | n.s. |
| **Parent education** | n.s. | n.s. | n.s. | n.s. |
| **Type of screen time activity** |  |  |  |  |
| AAC | n.s. | n.s. | n.s. | n.s. |
| Connecting with friends/family | n.s. | n.s. | n.s. | n.s. |
| Connecting with therapists | n.s. | 0.3 (0.04, 0.93),  p=0.05 ^§^ | n.s. | 0.6 (0.34, 0.87),  p=0.011 ^†^ |
| Playing educational games | 0.8 (0.58, 0.97), p=0.035 ^†^ | n.s. | n.s. | n.s. |
| Listening to music | n.s. | n.s. | n.s. | n.s. |
| Reading books | n.s. | n.s. | n.s. | n.s. |
| Reading the news | 0.6 (0.33, 0.99),  p=0.045 ^§^ | n.s. | n.s. | n.s. |
| School work/online learning | n.s. | n.s. | n.s. | n.s. |
| Social media | n.s. |  |  |  |
| Playing therapeutic apps | n.s. | n.s. | n.s. | n.s. |
| Playing video games | 0.8 (0.74, 0.95),  p=0.008 ^†^  0.8 (0.75, 0.94),  p=0.003 ^§^ | 0.9 (0.77, 0.98),  p=0.019 ^§^ | 0.9 (0.75, 0.96),  p=0.011 ^†^  0.8 (0.75, 0.94),  p=0.002 ^§^ | n.s. |
| Watching videos | 0.9 (0.76, 0.98),  p=0.028 ^†^  0.8 (0.74, 0.93),  p=0.002 ^§^ | 0.9 (0.76, 0.98),  p=0.030 ^§^ | 0.8 (0.75, 0.96),  p=0.008 ^§^ | n.s. |
| ^†^ Weekday use  ^§^ Weekend use | | | | |

####

####
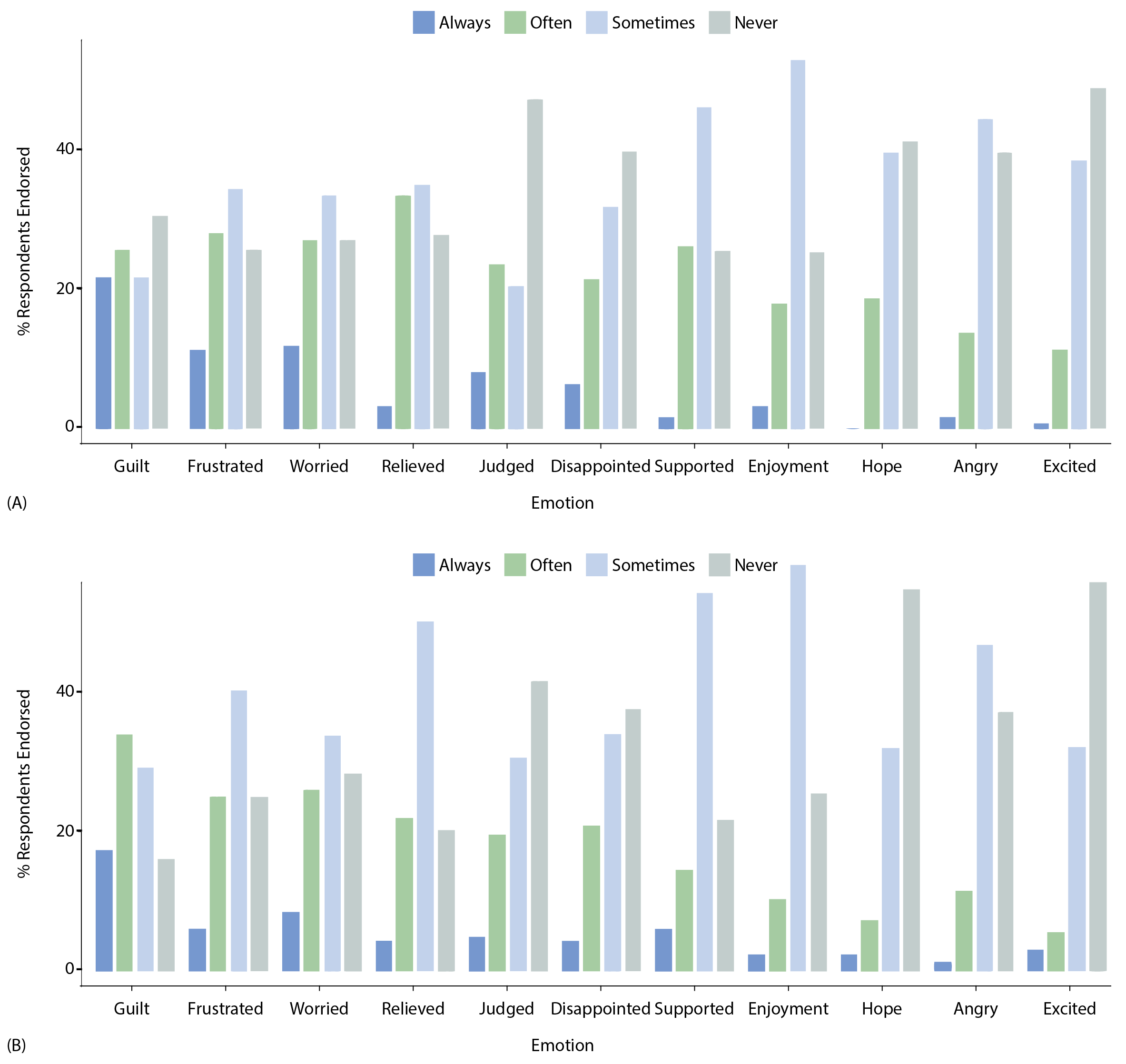


#### **Supplementary Figure 1.** Parental emotions associated with children’s screen time for the (A) ASD and (B) community sample groups.

####

#### **Supplementary Methods 1.** Survey instrument.

**ABOUT TECHNOLOGIES YOUR CHILD USES SINCE SCHOOLS CLOSED DUE TO COVID-19**

1. Before school closures due to COVID-19, how many hours per day did your child use screen technology (for example: TV, computers/laptops, game consoles or handheld consoles, smartphones, tablets)? Please estimate an average per day.

Weekdays ____hours/day Weekends____hours/day

2. Since school closures due to COVID-19, how many hours per day does your child use screen technology (for example: TV, computers/laptops, game consoles or handheld consoles, smartphones, tablets)? Please estimate an average per day.

Weekdays ____hours/day Weekends____hours/day

3. Since school closures due to COVID-19, how many hours per day does your child spend using the following devices on weekdays? Please estimate an average per day.

TV__________(hours/day)

Computer/laptop __________(hours/day)

Game consoles (examples: Nintendo, XBox, Playstation) __________(hours/day)

Smartphone (example: iPhone, Samsung Galaxy) __________(hours/day)

Tablet (example: iPad, Samsung Galaxy) __________(hours/day)

4. Since school closures due to COVID-19, how many hours per day does your child spend using the following devices on weekends? Please estimate an average per day.

TV__________(hours/day)

Computer/laptop __________(hours/day)

Game consoles (examples: Nintendo, XBox, Playstation) __________(hours/day)

Smartphone (example: iPhone, Samsung Galaxy) __________(hours/day)

Tablet (example: iPad, Samsung Galaxy) __________(hours/day)

**WHAT YOUR CHILD DOES WITH TECHNOLOGY SINCE SCHOOLS CLOSED DUE TO COVID-19**

5. Since schools closed due to COVID-19, how many hours per day does your child use technology for the following activities on weekdays?

Augmentative and alternative communication (example: Proloquo2Go) __________(hours/day)

Connecting with other socially (examples: using FaceTime, Zoom, Skype, WhatsApp)__________(hours/day)

Connecting with therapists or service providers (examples: using Zoom or OTN)__________(hours/day)

Educational apps (examples: Khan Academy, Epic, Prodigy, Raz Kids, IXL, Kahoot)__________(hours/day)

School work/Online learning (example: Google Classroom) __________(hours/day)

Listen to music (examples: Apple Music, Spotify) __________(hours/day)

Reading, watching, or listening to the news __________(hours/day)

Reading, watching or listening to books __________(hours/day)

Social media (examples: Facebook, Twitter, Instagram, TikTok) __________(hours/day)

Therapeutic apps__________(hours/day)

Video games for fun (example: Minecraft, Fortnite) __________(hours/day)

Watch videos (examples: YouTube, NetFlix) __________(hours/day)

6. Since schools closed due to COVID-19, how many hours per day does your child use technology for the following activities on weekends?

Augmentative and alternative communication (example: Proloquo2Go) __________(hours/day)

Connecting with other socially (examples: using FaceTime, Zoom, Skype, WhatsApp)__________(hours/day)

Connecting with therapists or service providers (examples: using Zoom or OTN)__________(hours/day)

Educational apps (examples: Khan Academy, Epic, Prodigy, Raz Kids, IXL, Kahoot)__________(hours/day)

School work/Online learning (example: Google Classroom) __________(hours/day)

Listen to music (examples: Apple Music, Spotify) __________(hours/day)

Reading, watching, or listening to the news __________(hours/day)

Reading, watching or listening to books __________(hours/day)

Social media (examples: Facebook, Twitter, Instagram, TikTok) __________(hours/day)

Therapeutic apps__________(hours/day)

Video games for fun (example: Minecraft, Fortnite) __________(hours/day)

Watch videos (examples: YouTube, NetFlix) __________(hours/day)

**HOW TECHNOLOGY IMPACTS YOUR FAMILY SINCE SCHOOLS CLOSED DUE TO COVID-19**

7. Overall, how has technology impacted your family’s quality of life since schools closed due to COVID-19? (pick one)

_____very positively

_____positively

_____neutral

_____negatively

_____very negatively

_____I don’t know

8. Overall, how has technology impacted your child’s quality of life since schools closed due to COVID-19? (pick one)

_____very positively

_____positively

_____neutral

_____negatively

_____very negatively

_____I don’t know

9. Overall, how has technology impacted your child’s mental health since schools closed due to COVID-19? (Pick one)

_____very positively

_____positively

_____neutral

_____negatively

_____very negatively

_____I don’t know

10. Since schools closed due to COVID-19, does your child lose time doing any of the activities below because s/he wants to use technology? (pick one for each activity)

Sleep

_____always _____often _____sometimes _____never _____I don’t know

Homework

_____always _____often _____sometimes _____never _____I don’t know

Physical activity

_____always _____often _____sometimes _____never _____I don’t know

Interacting with others

_____always _____often _____sometimes _____never _____I don’t know

11. Since schools closed due to COVID-19, in which of these areas do you feel your child has benefited most from technology? (pick all that apply)

_____Coping with social isolation/connecting with others

_____Social skills

_____Motor skills

_____Emotion regulation

_____Education/online schooling

_____Leisure/recreation

_____Mental Health

_____My child has not benefited from technology in any area

_____Other -please specify ___________________________

_____I don’t know

12. Since schools closed due to COVID-19, in which of these areas do you feel your family has benefited most from technology? (pick all that apply)

____Home education

____Help with child minding

____Coping with social isolation/connecting with others

____Leisure/recreation

____Using technology as a reward

____We have not benefited from technology in any area

____I don’t know

____Other – please specify_______________________

13. Since schools closed due to COVID-19, how often do you experience these feelings when your child uses technology? (pick one for each category)

Angry

_____always _____often _____sometimes _____never

Enjoyment

_____always _____often _____sometimes _____never

Excited

_____always _____often _____sometimes _____never

Hope

_____always _____often _____sometimes _____never

Supported

_____always _____often _____sometimes _____never

Relieved

_____always _____often _____sometimes _____never

Guilt

_____always _____often _____sometimes _____never

Judged

_____always _____often _____sometimes _____never

Frustrated

_____always _____often _____sometimes _____never

Disappointed

_____always _____often _____sometimes _____never

Worried

_____always _____often _____sometimes _____never

Other - please specify ___________________________

_____always _____often _____sometimes _____never

**ABOUT YOUR CHILD**

14. When was your child born? (mm/yy) (example: if they were born in April 1980, please enter 04/1980) (optional) _____

15. What is your child’s gender? _____ Male _____ Female _____ other

16. How many children live in your home? Please include the child you are filling out this survey for _____ (number of children in household)

17. Which of these options best describes your child’s racial background? (pick all that apply)

_____Black (examples: Ethiopian, Jamaican, Kenyan, Nigerian, Somalian, Vincentian)

_____East Asian (examples: Chinese, Japanese, Korean)

_____First Nations/Metis/Inuit

_____Latin American (examples: Columbian, Cuban, Mexican)

_____Middle Eastern (examples: Afghani, Iranian, Lebanese, Saudi Arabian, Syrian)

_____South Asian (examples: Bangladeshi, Indian, Pakistani)

_____Southeast Asian (examples: Filipino, Malaysian, Thai)

_____White (examples: European)

_____Other -please specify ___________________________

_____Prefer not to say

18. Does your child have a primary diagnosis of any of these conditions? (pick one)

_____Autism spectrum disorder (ASD)

_____Attention deficit/hyperactivity (ADHD)

_____Anxiety disorder

_____Intellectual disability

_____Learning Disability

_____Obsessive compulsive disorder

_____Other diagnosis -please specify ___________________________

_____No diagnosis

19. Does your child have any other symptoms or conditions? (pick all that apply)

_____Autism spectrum disorder (ASD)

_____Attention deficit/hyperactivity (ADHD)

_____Anxiety disorder

_____Depression

_____Epilepsy

_____Intellectual disability

_____Learning Disability

_____Obsessive compulsive disorder

____ Attention difficulties

____ Aggressive behaviour (towards others)

____ Self-injurious behaviours

____ Sleep difficulties

_____Other diagnosis -please specify ___________________________

_____No diagnosis

20. Did your child receive specialized support at school before schools closed due to COVID-19? (pick all that apply)

_____My child attends a regular classroom

_____My child receives additional support in a mainstream classroom

_____My child is in a special education classroom for at least half the day (example: Intensive Support Program (ISP))

_____My child is in a gifted program

_____My child does not attend school

_____My child is home-schooled

_____Other - please specify: _____________________

21. How does your child communicate with others most of the time? (pick all that apply)

_____My child does not communicate verbally

_____My child uses augmentative and alternative communication (AAC) (examples: PECS, Proloquo)

_____My child uses sign language

_____My child writes or types words on a computer

_____My child doesn’t have words, but uses non-verbal cues to communicate (examples: gestures, facial expressions)

_____My child uses verbal communication (speaks words and sentences)

_____Other - please specify ___________________________

**ABOUT YOU AND YOUR FAMILY**

22. What is your relationship to your child? (pick one)

_____Mother

_____Father

_____Stepfather

_____Stepmother

_____Grand mother

_____Grand father

_____Foster mother

_____Foster father

_____Other -please specify ___________________________

23. During the COVID-19 closures, how many adults in your household help with childcare? __________

24. During the COVID-19 closures, how many of the adults in your household who help with childcare are working from home? ____________

25. During the COVID-19 closures, how many of the adults in your household who help with childcare leave home for work at least one day per week? __________

26. What is the total household income of your family for the year? (pick one)

_____Less than $30,000

_____$30,000-$49,0000

_____$50,000-$74,999

_____$75,000-$99,000

_____More than $100,000

_____Prefer not to say

27. What is the highest level of education completed in your household? (pick one)

_____Elementary school

_____Secondary school

_____College

_____University

_____Other - please specify ___________________________

_____None

_____Prefer not to say

28. Please enter the first three digits of your postal code (example: if your postal code is X1Y 2Z3, only enter X1Y) (optional) _____

29. Is there anything else you would like to share about how your child uses technology? For example, anything that you think is especially helpful or frustrating that we have not asked about in the previous questions.

_______________________________________________________________________
